# Tinosporaside from *Tinospora cordifolia* Encourages Skeletal Muscle Glucose Transport through Both PI-3-Kinase- and AMPK-Dependent Mechanisms

**DOI:** 10.3390/molecules28020483

**Published:** 2023-01-04

**Authors:** Akansha Mishra, Khushbu Sharma, Jyotsana Pandey, Kapil Dev, Sleman Kadan, Mahendra Sahai, Ishbal Ahmad, Arvind K. Srivastava, Akhilesh K. Tamrakar, Hilal Zaid, Rakesh Maurya

**Affiliations:** 1 Division of Biochemistry, CSIR-Central Drug Research Institute, Lucknow 226031, India; 2 Academy of Scientific and Innovative Research (AcSIR), Ghaziabad 201002, India; 3 Department of Medicinal Chemistry, Institute of Medical Sciences, Banaras Hindu University, Varanasi 221005, India; 4 Medicinal and Process Chemistry Division, CSIR-Central Drug Research Institute, Lucknow 226031, India; 5 Qasemi Research Center, Al-Qasemi Academic College, P.O. Box 124, Baqa El-Gharbia 30100, Israel; 6 Faculty of Medicine, Arab American University, Jenin P.O. Box 240, Palestine

**Keywords:** tinosporaside, antihyperglycemic activity, glucose utilization, db/db mice

## Abstract

The stem of *Tinospora cordifolia* has been traditionally used in traditional Indian systems of medicine for blood sugar control, without the knowledge of the underlying mechanism and chemical constitution responsible for the observed anti-diabetic effect. In the present study, Tinosporaside, a diterpenoid isolated from the stem of *T. cordifolia*, was investigated for its effects on glucose utilization in skeletal muscle cells, which was followed by determining the anti-hyperglycemic efficacy in our diabetic db/db mice model. We found that tinosporaside augmented glucose uptake by increasing the translocation of GLUT4 to the plasma membrane in L6 myotubes, upon prolonged exposure for 16 h. Moreover, tinosporaside treatment significantly increased the phosphorylation of protein kinase B/AKT (Ser-473) and 5′ AMP-activated protein kinase (AMPK, Thr-172). These effects were abolished in the presence of the wortmannin and compound C. Administration of tinosporaside to db/db mice improved glucose tolerance and peripheral insulin sensitivity associated with increased gene expression and phosphorylation of the markers of phosphoinositide 3-kinases (PI3Ks) and AMPK signaling in skeletal muscle tissue. The findings revealed that tinosporaside exerted its antidiabetic efficacy by enhancing the rate of glucose utilization in skeletal muscle, mediated by PI3K- and AMPK-dependent signaling mechanisms.

## 1. Introduction

Type 2 diabetes mellitus (T2DM) is a catastrophic metabolic disorder and continues to be a major risk for morbidity and mortality throughout the world [1]. It is characterized by impaired glucose and lipid metabolism in insulin-sensitive tissues, mainly the skeletal muscle, liver and adipose tissue, leading to establishment of persistent hyperglycemia, the hallmark of diabetes [2,3]. Skeletal muscle is the principal site of postprandial glucose disposal and is a major determinant of insulin sensitivity. Glucose uptake in skeletal muscle is majorly mediated by the insulin-responsive glucose transporter 4 (GLUT4), and is a major regulatory process in the homeostatic control of blood glucose levels [4]. The activity of glucose transporters has long been established to be mainly regulated by two distinct signaling mechanisms: the phosphatidylinositol-3-kinase(PI3K)-mediated pathway, responsible for insulin-stimulated glucose utilization, and the 5′-AMP-activated protein kinase(AMPK)-mediated pathway, involved in exercise-driven stimulation of glucose utilization [5,6,7,8]. Hence, augmenting glucose uptake into skeletal muscles through any of these pathways may regulate glucose homeostasis for the better management of type 2 diabetes mellitus.

**Figure 1:**
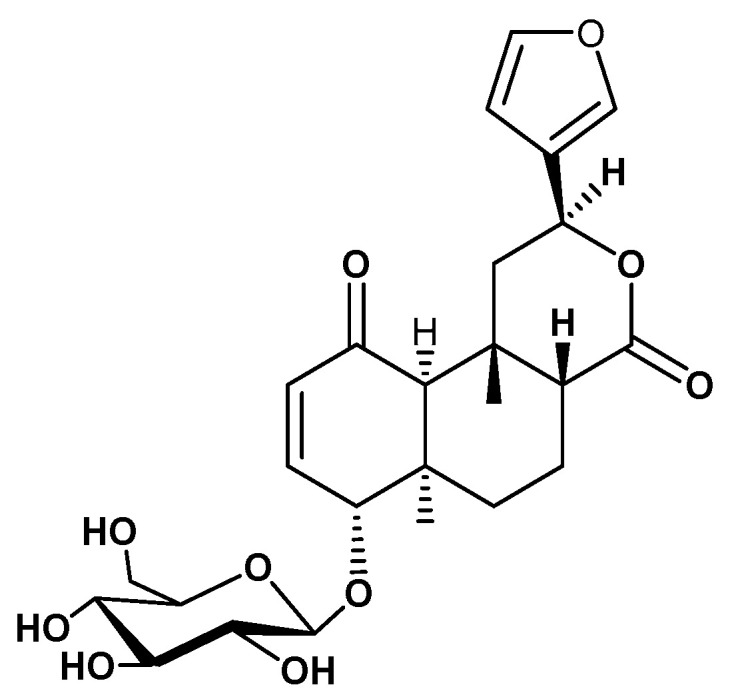
Chemical structure of tinosporaside, isolated from the stem of *Tinospora cordifolia*.

Current medications for diabetes are not addressing the long-term glycemic control in type 2 diabetic patients. Therefore, research towards finding potential antidiabetic agents from herbal or natural plant resources, that can be used as treatment regimen, are urgently needed. *Tinospora cordifolia* (Willd.) Miers ex Hook. f. & Thomson (Family: Menispermaceae), commonly known as giloe or guduchi in Hindi, is a dioecious creeper with significant medicinal properties [9]. It is designated as a Rasayana drug in Ayurveda and recommended for treatment of various human ailments, including viral infections, kidney disorders, inflammation, neural disorders, hyperglycemia, dyslipidemia, etc. [10,11,12,13,14,15,16]. The plant is reported to contain bioactive phytochemicals, including diterpenoid lactones, sesquiterpenoids, glycosides, alkaloids, steroids, and phenolics [9,16]. The stem of *T. cordifolia* has been used in the traditional Indian system of medicine for controlling blood sugar levels for centuries, without knowing much about its mechanisms of action [17,18]. The extract of the plant has been shown to stimulate glucose uptake in a tumor cell model system [19]. However, the chemical constituents responsible for its antidiabetic action and the underlying mechanism of action have yet to be well established. The present study describes that tinosporaside (Figure 1), a bioactive molecule isolated from the stem of *T. cordifolia*, stimulates glucose uptake in skeletal muscle cells via activating PI3K- and AMPK-dependent signaling pathways.

## 2. Results

### 2.1. Tinosporaside Augments Glucose Uptake in L6 Cells

**Figure 2:**
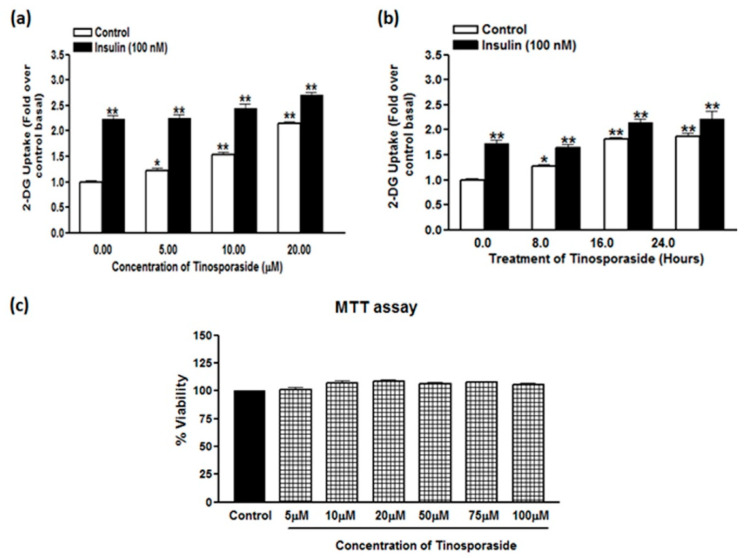
Dose- and time-dependent effect of tinosporaside on glucose uptake. (**a**) L6 myotubes were treated with different concentrations of tinosporaside for 16 h. After incubation, a subset of cells were stimulated with 100 nM of insulin for 20 min, which was followed by the determination of 2-DG uptake. (**b**) L6 myotubes were treated at 10 µM concentration of tinosporaside for a different time period and 2-DG uptake was determined in absence (white bars) or presence of 100 nM insulin (black bars). (**c**) L6 cells were treated with tinosporaside for 24 h at different concentrations, from 5 µM to 100 µM, and cytotoxicity was measured using the MTT assay. Results are expressed as fold stimulation over control basal. Results shown are mean ± SE of three independent experiments. * *p* < 0.05, ** *p* < 0.01, relative to control basal.

Pretreatment of L6 myotubes with tinosporaside enhanced the rate of glucose uptake in a dose- and time-dependent manner (Figure 2a,b). Tinosporaside enhanced basal glucose uptake at a minimum concentration of 5.0 μM (1.22-fold, *p* < 0.05 vs. control) with sustained stimulation that reached its maximum at a 20 μM concentration (2.15-fold, *p* < 0.01 vs. control), following treatment for 16 h (Figure 2a). The significant activity of glucose uptake stimulation was observed at a 10 μM concentration, so this concentration was selected for further experimentation. Tinosporaside significantly increased glucose uptake in L6 myotubes, starting from a treatment time of 8 h, and reached its maximum at 16 h, with sustained stimulation up to 24 h (Figure 2b). As the maximum stimulation was observed at 16 h, further experimentation was performed with this treatment duration. Further, the effect of tinosporaside on insulin-stimulated glucose uptake was studied upon acute insulin exposure (100 nM for 20 min). Insulin alone caused a significant change in glucose uptake (2.23-fold, *p* < 0.01 vs. control basal) in L6 myotubes (Figure 2a), whereas pre-treatment with tinosporaside did not induce any significant effect on the insulin response to stimulate glucose uptake at lower concentrations, but produced an additive effect at 20 μM concentration (Figure 2a,b). Findings indicated the involvement of some additional mechanism distinct from insulin signaling in tinosporaside-mediated glucose uptake stimulation. Of note, overnight treatment with tinosporaside had no significant effect on the viability of L6 cells at concentration ranged up to 100 μM, as determined using the MTT assay (Figure 2c).

### 2.2. Effect of Tinosporaside on GLUT4 Translocation in L6-GLUT4myc Myotubes

**Figure 3:**
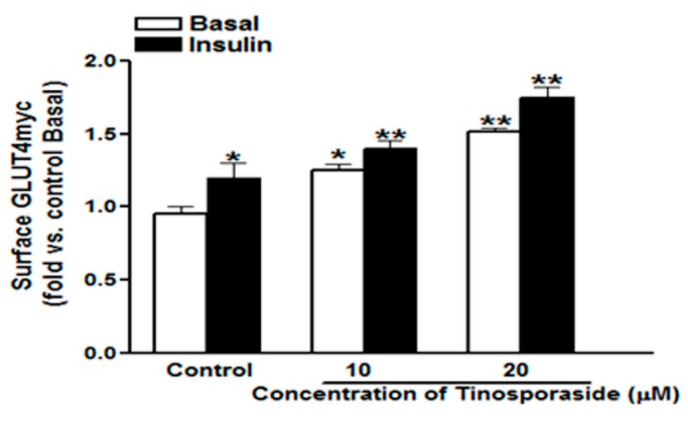
Dose-dependent effect of tinosporaside on GLUT4*myc* translocation in L6-GLUT4*myc* myotubes. Cells were incubated with 10 μM and 20 μM concentrations of tinosporaside for 16 h, with the final three hours in a serum-deprived medium. After incubation, myotubes were left untreated (white bars) or stimulated with 100 nM insulin (black bars) for 20 min, which was followed by the determination of the proportion of GLUT4*myc* at the cell surface. Results are expressed as fold stimulation over control basal. Results shown are mean ± SE of three independent experiments, each performed in triplicate. * *p* < 0.05, ** *p* < 0.01, relative to respective control condition.

The glucose uptake stimulation in skeletal muscle is mainly ascribed to the enhanced translocation of insulin-sensitive glucose transporter4 (GLUT4) from intracellular compartments to cells periphery, to facilitate the entry of glucose inside the cell [20]. Therefore, the level of GLUT4 present at the cell surface determines the rate of uptake and its further utilization. The overnight treatment with tinosporaside enhanced the surface level of GLUT4 in a dose-dependent manner, with a fold stimulation of 1.32 and 1.52 (*p* < 0.05, *p* < 0.01 vs. control basal) at the 10 μM and 20 μM concentrations, respectively (Figure 3). In control cells, acute treatment with insulin (100 nM for 20 min) raised the surface GLUT4*myc* level by 1.2-fold (*p* < 0.05 vs. control basal). In cells pretreated with tinosporaside (20 μM), the insulin response was significantly potentiated, as compared with control insulin. Findings verified the effect of tinosporaside to stimulate glucose utilization in skeletal muscle cells (Figure 3).

### 2.3. Effect of Wortmannin on Tinosporaside-Stimulated Glucose Uptake in L6 Myotubes

**Figure 4:**
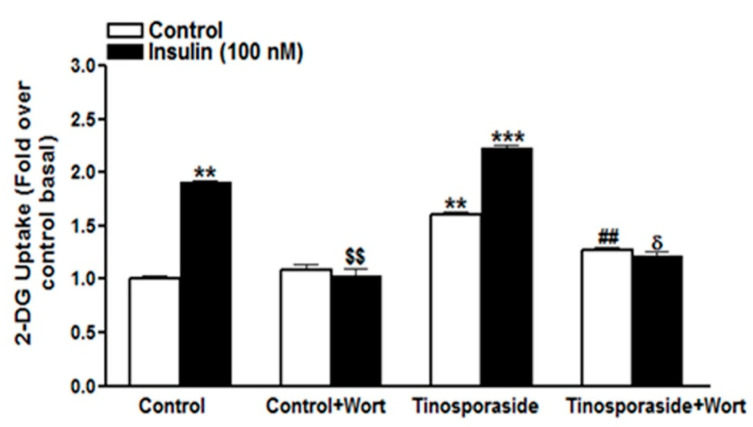
Effect of wortmannin on tinosporaside-induced glucose uptake in L6 myotubes: Cells were incubated in the absence (Control) or the presence of tinosporaside (10 μM) for 16 h without or with wortmannin (100 nM). After incubation, myotubes were left untreated (white bars) or stimulated with 100 nM insulin (black bars) for 20 min, which was followed by the determination of the glucose uptake. Results are expressed as fold stimulation over control basal and performed in triplicate. Results shown are mean ± SE of three independent experiments. ** *p* < 0.01, *** *p* < 0.001, relative to control basal; ^$$^ *p* < 0.01, relative to control insulin-treated group; ^##^ *p* < 0.01, relative to basal tinosporaside-treated group; ^δ^ *p* < 0.05 relative to tinosporaside insulin-treated group.

To investigate the mechanism by which tinosporaside augmented glucose uptake in L6 myotubes, first we assessed its response in presence of wortmannin, a specific inhibitor of the PI3K. The presence of wortmannin (100 nM) completely reversed the insulin-induced glucose uptake to basal level. Similarly, tinosporaside-induced glucose uptake was inhibited in the presence of wortmannin in L6 myotubes (Figure 4). Tinosporaside-mediated potentiation of insulin response to increase glucose uptake was also completely abolished to the basal level in the presence of wortmannin (Figure 4). These results indicate that tinosporaside stimulates glucose uptake in L6 myotubes in part via the PI3K signaling pathway.

### 2.4. Effect of Compound C on Tinosporaside-Stimulated Glucose Uptake in L6 Myotubes

**Figure 5:**
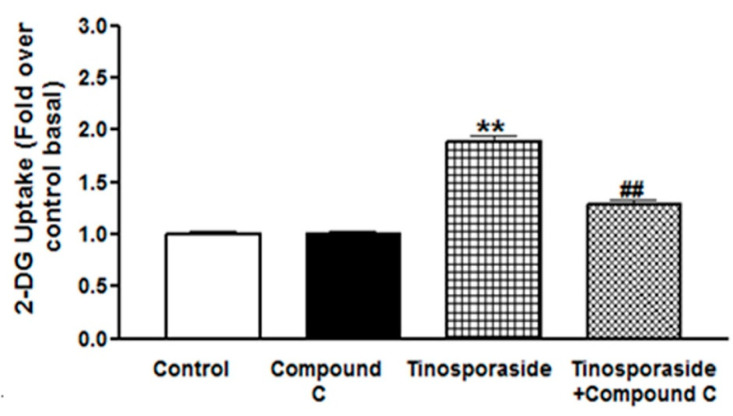
Effect of Compound C on tinosporaside-induced glucose uptake in L6 myotubes. L6 myotubes were treated with tinosporaside in the absence or presence of compound C for 30 min, which was followed by the determination of 2-DG uptake. Results shown are mean ± SE of three independent experiments. ** *p* < 0.01, relative to control; ^##^ *p* < 0.01, relative to the tinosporaside treated group.

Activation of AMPK has been known to contribute to an increase in glucose uptake and utilization in L6 myotubes [21]. Hence, to investigate the effect of tinosporaside on the activation of the AMPK pathway, we treated the L6 cells with tinosporaside in the presence of Compound C, a specific inhibitor of the AMPK pathway and studied the effect on glucose uptake. Tinosporaside significantly stimulated glucose uptake to 1.7-fold over basal, and this effect was inhibited in the presence of Compound C (*p* < 0.01 vs. tinosporaside-treated group, Figure 5). These results suggest that the effect of tinosporaside on glucose transport is mediated in part by activation of the AMPK-mediated signaling route.

### 2.5. Antidiabetic Effect of Tinosporaside on C57BL-Ks db/db Mice

**Figure 6:**
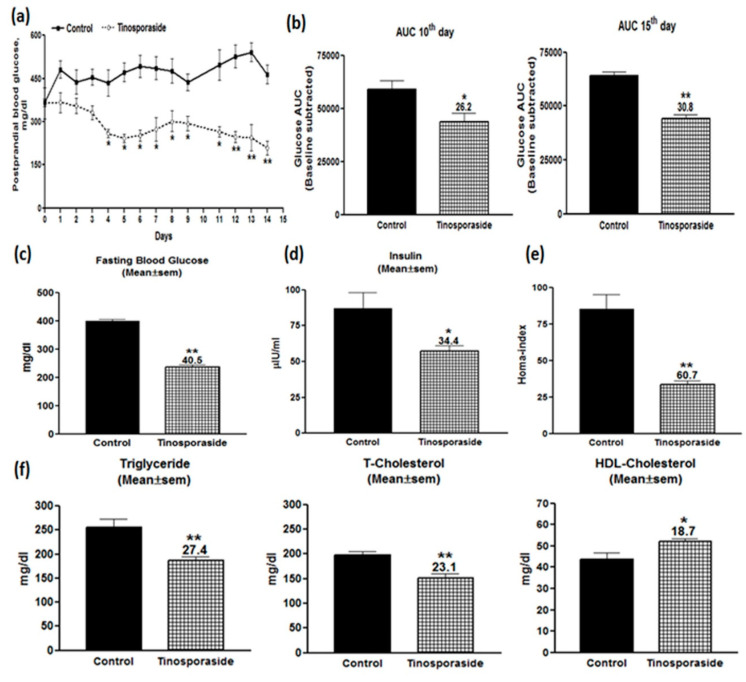
Antidiabetic effect of tinosporaside in C57BL-Ks db/db mice. Mice were treated daily with tinosporaside for15 consecutive days and blood glucose levels were monitored daily. Shown above are effects on postprandial blood glucose levels (**a**), OGTT at day 10 and day 15 (**b**), fasting blood glucose (**c**), serum insulin level (**d**), HOMA-index (**e**), and serum lipid profile (**f**). Data are expressed as the mean ± SE value, *n* = 5. * *p* < 0.05, ** *p* < 0.01, relative to control animals.

Given the *in vitro* effect of tinosporaside to stimulate glucose utilization in skeletal muscle cells, we further investigated it for *in vivo* antidiabetic activities in a genetically diabetic db/db mice model system. Tinosporaside was administered through oral gavages for 15 consecutive days at a 30 mg/kg dose. Figure 6a showed the effect of tinosporaside on postprandial blood glucose profile of db/db mice during the 15 days of uninterrupted dosing. It was found that tinosporaside treatment decreased the blood glucose level in a temporal manner; a significant decrease in blood glucose level was evident from day 4, and the effect persisted until the end of the experiment. To investigate the effect of tinosporaside on improvements in glucose tolerance, the oral glucose tolerance test (OGTT) was carried out on days 10 and 15 of the experiment. Figure 6b shows that, on day 10, tinosporaside effectively repelled the rise in postprandial hyperglycemia post-glucose challenge; an overall improvement in the glucose levels’ AUC from 0 to 120 min was calculated to be around 26.2% (*p* < 0.05). Meanwhile, on day 15, it was found that tinosporaside effectively inhibited the rise in postprandial hyperglycemia, post-glucose challenge (Figure 6b). The improvement in glucose AUC from 0 to 120 min from tinosporaside was calculated to be around 30.8% (*p* < 0.01), as compared with the vehicle-treated control group.

Further, serum samples from tinosporaside-treated db/db mice were investigated to evaluate the effect on fasting blood glucose and serum insulin levels. It was found that tinosporaside significantly improved the fasting blood glucose by 40.5% (*p* < 0.01, Figure 6c), while a decline of 34.4% (*p* < 0.05) in serum insulin level was observed in the tinosporaside-treated group (Figure 6d) at day 15 of experiment. The improvement in the fasting blood glucose and fasting serum insulin profile can eventually lead to improvement of insulin resistance, which was reflected in our study in the homeostatic model assessment (HOMA) index. It was found that tinosporaside significantly lowered the HOMA-index to the tune of 60.7% (*p* < 0.01), as shown in Figure 6e. Thus, the improvement of fasting blood glucose by tinosporaside refers to improvements in insulin sensitivity in db/db mice. The serum lipid profile of db/db mice after 15 days of repeated dosing with tinosporaside was examined to observe its additional anti-dyslipidemic effects. Tinosporaside decreased the serum triglycerides (TG) by 27.4% (*p* < 0.01), total cholesterol (T-Chol) by 23.1% (*p* < 0.01), and enhanced the level of serum high-density lipoproteins (HDL-C) by 18.7% (*p* < 0.05), respectively (Figure 6f).

### 2.6. Effect of Tinosporaside on Gene Expression in the Skeletal Muscle of db/db Mice

**Figure 7:**
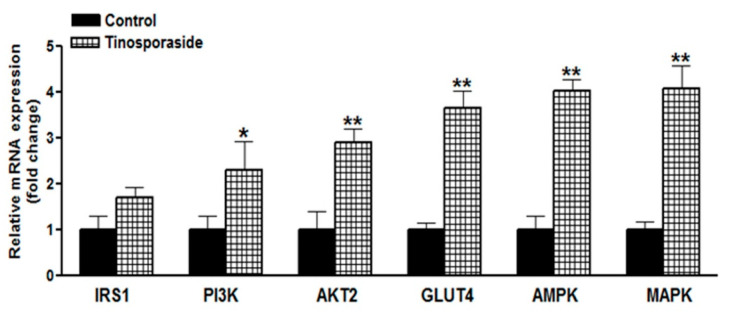
Effect of tinosporaside on gene expression of diabetes-related genes in muscle tissues of C57BL-Ks db/db mice. Skeletal muscle tissues were excised, RNA was isolated from the control and tinosporaside-treated mice, which was followed by real-time PCR for determination of the expression profiling of the indicated genes. Results are expressed as the mean ± SE, *n* = 3. * *p* < 0.05, ** *p* < 0.01, relative to control group.

Given the role of PI3K and AMPK pathways in tinosporaside-mediated glucose utilization in L6 cells, we further assessed the effect on expression of key genes involved in the PI3K and AMPK signaling pathways in skeletal muscle tissue collected from db/db mice treated with tinosporaside. Based on the results of the real-time PCR, we found a significant difference in the expression profile of genes related to the insulin-dependent and insulin-independent pathways, between tinosporaside-treated and control groups (Figure 7). It was observed that the expression of insulin receptor substrate (IRS-1), thymoma viral proto-oncogene 2 (AKT2), phosphatidlyinositol-3 kinase catalytic subunit (PIK3CG) and GLUT4 were all involved in the insulin signaling pathway, and were markedly upregulated in tinosporaside-treated mice. Tinosporaside also upregulated the expression of AMPK and MAPK, which enhance the translocation of GLUT4 via an insulin-independent mechanism, mediated through activation of AMPK pathway. These findings verified the participation of both PI3K- and AMPK-driven mechanisms in tinosporaside-mediated regulation of glucose metabolism in the skeletal muscle of db/db mice.

### 2.7. Effect of Tinosporaside on the Protein Levels of the PI3K and AMPK Signaling Pathways in Skeletal Muscle of db/db Mice

**Figure 8:**
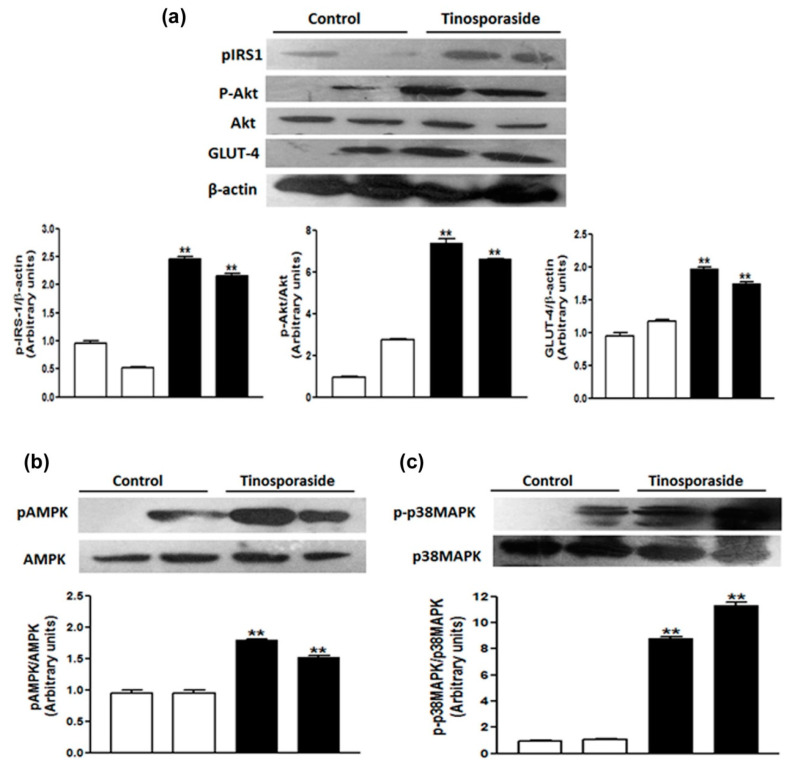
Tinosporaside-stimulated glucose uptake is mediated by activation of PI3K/AKT- and AMPK-dependent pathways. Western blot analysis of phospho-IRS-1, phospho-Akt, Akt and GLUT4 (**a**), phospho-AMPK (**b**) and p38 MAPK (**c**) in skeletal muscle of db/db mice treated with vehicle (white bars) or tinosporaside (Black bars) are presented. Results are expressed as the mean ± SE. ** *p* < 0.01, relative to control condition.

We further validated the activation of the PI3K and AMPK signaling pathways underlying the anti-hyperglycemic and anti-dyslipidemic effects of tinosporaside at the protein level using western blot analysis of skeletal muscle tissues from db/db mice. It was found that tinosporaside significantly stimulated the phosphorylation of IRS-1 and AKT2, which are a critical node of insulin signaling (Figure 8) in skeletal muscle. The phosphorylation of AMPK and its downstream target p-38 MAPK at key activation sites was also increased in the muscle tissue of the tinosporaside-treated group, relative to control mice (Figure 8). Furthermore, tinosporaside increased the expression of GLUT4 protein in skeletal muscle of db/db mice These *in vivo* results are in accordance with the *in vitro* data, suggesting that tinosporaside treatment improved the glucose utilization in skeletal muscle via the activation of PI3K- and AMPK-dependent signaling pathways, leading to improved glucose homeostasis in db/db mice.

## 3. Discussion

One of the major defects in type 2 diabetes mellitus is altered glucose transport, associated with defective GLUT4 translocation and impairment in the insulin signaling cascade [22]. In the present study, we addressed the molecular effects of tinosporaside on glucose utilization in skeletal muscle cells, along with the *in vivo* antihyperglycemic effect in the diabetic db/db mice model. Tinosporaside treatment strongly stimulated the rate of glucose uptake in L6 myotubes under both the basal as well as insulin-stimulated state in a concentration- and time-dependent manner, suggesting that tinosporaside improved the glucose utilization in skeletal muscle cells. Glucose uptake in skeletal muscle can be attributed to translocation of insulin-sensitive GLUT4 from the intracellular vesicle to the cell membrane, where they facilitate the entry of glucose inside the cells [4]. Here, tinosporaside treatment induced a concentration-dependent increase in the surface level of GLUT4 in L6-GLUT4*myc* myotubes, verifying that tinosporaside encouraged the translocation and redistribution of GLUT4 to the plasma membrane, leading to enhanced glucose uptake inside the cells. It has been demonstrated that insulin-stimulated muscle glucose uptake is highly susceptible to insulin resistance attributed to impaired GLUT4 translocation, and stimulating GLUT4 translocation is helpful to maintain glucose homeostasis [23]. The *in vitro* effects of tinosporaside in augmenting GLUT4 translocation suggests its potential in regulating glucose homeostasis.

Therefore, the effects of tinosporaside to regulate glucose homeostasis under *in vivo* conditions were further investigated in genetically diabetic db/db mice, a well-characterized model of type 2 diabetes mellitus [24]. Treatment with tinosporaside showed a significant lowering of blood glucose levels and significant improvement in glucose tolerance in db/db mice, verifying the efficacy of tinosporaside to regulate glucose metabolism in diabetic conditions presented by db/db mice model. The beneficial effects of tinosporaside were associated with improvement in fasting blood glucose levels, serum insulin levels, and HOMA-index. Moreover, tinosporaside treatment improved the lipid profile in db/db mice, suggesting the therapeutic potential of tinosporaside for the management of insulin resistance associated with type 2 diabetes. Based on the *in vitro* effects of tinosporaside to regulate glucose utilization in skeletal muscle cells, we assume that the blood glucose-lowering effect of tinosporaside might be attributed to improved insulin sensitivity, leading to increased glucose utilization in peripheral tissues.

In skeletal muscle, the enhanced rate of glucose uptake is attributed to two major signaling pathways: one is the PI3K-dependent signaling cascade, responsible for insulin action, and another is the AMPK-mediated insulin-independent pathway [25]. Insulin regulates glucose transport by activating insulin receptor substrate-1 dependent PI3K pathway, which activates PKB/Akt and its downstream protein targets, such as AS160, leading to enhanced translocation of GLUT4 to cell membrane [26,27]. The tinosporaside-stimulated glucose transport in L6 myotubes was completely abolished in the presence of wortmannin, a chemical inhibitor of PI3K [28], suggesting that tinosporaside activates the PI3K pathway. Activated PI3K conveyed the signal through activation of its downstream signaling node i.e., Akt. When the Akt pathway was blocked, tinosporaside-stimulated glucose transport was markedly reduced. In agreement with the *in vitro* studies, upregulated expression of genes, as well as the activation of IRS-1, PI3K, p-Akt, and GLUT4 by tinosporaside in skeletal muscle tissues of db/db mice, verified that tinosporaside exerted its effect by enhancing signal transduction through a PI3K-dependent pathway. Moreover, the presence of compound C, a pharmacological inhibitor of AMPK pathway, also effectively inhibited the tinosporaside-stimulated glucose uptake in L6 myotubes, suggesting the potential role of AMPK-driven signaling in tinosporaside-induced glucose uptake in L6 myotubes. The results were also confirmed by the *in vivo* data, where tinosporaside effectively phosphorylated AMPK and its downstream target p38 MAPK, and increased the expression of GLUT4 protein in skeletal muscle of db/db mice. Activation of AMPK could recruit AS160, which further contributes to stimulation of GLUT4 translocation and the subsequent increase in the rate of glucose uptake in skeletal muscle [29]. Several naturally-occurring compounds like berberine [30,31,32], tangeretin [33], resveratol [34], and dietary phytoestrogens [35] have been reported to activate the AMPK pathway and improve the glucose and lipid homeostasis in *in vivo* models. Taken together, our findings provide adequate evidence for the idea that anti-hyperglycemic potential of tinosporaside is associated with enhanced glucose utilization in skeletal muscle, mediated through activation of both PI3K- and AMPK-dependent signaling pathways.

## 4. Material and Method

### 4.1. Materials

Dulbecco’s Modified Eagle Medium (DMEM), fetal bovine serum (FBS), trypsin, and antibiotic/antimycotic solution were procured from Gibco, Waltham, MA, USA. 2-deoxyglucose, polyclonal anti-*myc*, monoclonal anti-actinin-1, and all other chemicals unless otherwise noted were from Sigma Chemical (St. Louis, MO, USA). 2-Deoxy-d-[^3^H]-glucose (2-DG) was from Perkin Elmer, CT, USA. Antibodies to phospho-IRS-1 (Tyr-895), phospho-Akt (Ser-473), Akt, GLUT4 (IF8), AMPKα, phospho-AMPKα (Thr-172) and β-actin were from Cell Signaling Technology (Danvers, MA, USA). Antibodies to phospho-p38 (Thr-180/Tyr-182) and p38 MAPK were from Santa Cruz Biotechnology (Dallas, TX, USA). Biochemical assay kits for the measurement of total triglyceride (TG), total cholesterol (TC) and high-density lipoprotein cholesterol (HDL-C) were procured from Dialab Chennai, India.

### 4.2. Plant Material

*Tinospora cordifolia* (Willd.) Miers (family: Menispermaceae) was collected from Banaras Hindu University, Varanasi, India, confirmed as *T. cordifolia* through comparison with the specimen kept in the herbarium of the Department of Medicinal Chemistry, Banaras Hindu University, Varanasi, India.

### 4.3. Extraction and Isolation of Tinosporaside

The powdered stem (2 kg) was heated with distilled water (3.5 L) over a water bath for 6 h, filtered. The aqueous extract was concentrated to 500 mL using a rotary evaporator. The aqueous extract was successively extracted with chloroform (3 × 500 mL) and *n*-butanol (3 × 500 mL) (SRL, Mumbai, India) to get corresponding chloroform (20 g) and *n*-butanol (38 g) soluble fractions, respectively. The *n*-butanol soluble (38 g) fraction was chromatographed over silica gel (60–120 mesh size) and eluted with the gradient polarity of methanol in chloroform and collected in ten fractions A1-A10. The fraction A3 (210 mg) was repacked over silica gel (230–400 mesh) and eluted with CHCl_3_: MeOH (93: 07) to yield tinosporaside (95 mg). The structure of tinosporaside (Figure 1) was established with the help of detailed spectroscopic data, and further confirmed with reports from literature [36,37].

### 4.4. Cell Culture

Experiments were performed in rat L6 skeletal muscle cells and L6 cells stably expressing rat GLUT4 with a *myc* epitope (L6-GLUT4*myc*). The L6-GLUT4*myc* cells were obtained as a kind gift from Dr. Amira Klip, Program in Cell Biology, The Hospital for Sick Children, Toronto, Canada. The Dulbecco’s Modified Eagle Medium (DMEM), supplemented with 10% FBS, was used for maintaining the cells under standard cell culture conditions. For differentiation to myotubes stage, cells were switched to DMEM with 2% FBS post confluence, for 4–5 days. Treatment was done in differentiated myotubes at different time points and concentrations as mentioned. For insulin-stimulation, cells were incubated for another 3 h in a serum-deprived medium, which was followed by treatment with insulin (100 nM) for 20 min [38].

### 4.5. Glucose Uptake Assay

Determination of 2-DG uptake in differentiated myotubes was performed as previously described [39]. Myotubes grown in 24-well plates were treated with tinosporaside, as indicated and glucose uptake was assessed for 5 min in HEPES-buffered saline containing 10 µM 2-DG (0.5 µCi/mL 2-[^3^H] DG) at room temperature. Cells were lysed and quantification of radioactivity incorporated inside the cells was performed using β-counter (Beckman Coulter, Brea, CA, USA). Glucose uptake in presence of cytochalasin B (50 µM) was considered as nonspecific and subtracted from all other values.

### 4.6. GLUT4 Translocation Measurement

Determination of GLUT4 translocation to the cell surface was performed in L6-GLUT4*myc* myotubes using an antibody-coupled colorimetric assay, as previously described [40]. L6-GLUT4*myc* myotubes were treated with tinosporaside for indicated time duration followed by fixing with 3% paraformaldehyde and quenching in 100 mM glycine. Then, plates were blocked with 5% FBS and incubated with primary anti-*myc* antibody for 1 h. After incubation, cells were washed thoroughly with PBS to remove excess labeling. After that, cell surface bound primary antibodies were probed with HRP-conjugated secondary antibodies, followed by washing with PBS. Amount of bound HRP was detected using O-phenylenediamine dihydrochloride reagent by measuring absorbance at 492 nm.

### 4.7. Cell Viability Assay

Cell viability was determined using 3-(4,5-dimethylthiazol-2-yl)-2,5-diphenyltetrazalium bromide (MTT) assay [41]. Cells were seeded in 48-well plates and allowed to attach for 24 h. Cells were treated with increasing concentrations of tinosporaside (5–100 μM) for 24 h. After that, 20 μL of MTT solution (5 mg/mL in PBS) was added into each well and incubated at 37 °C for 4 h. The absorbance of the resulting formazan product was measured at 540 nm using an ELISA plate reader (Molecular Devices, San Jose, CA, USA).

### 4.8. Animals

Male C57BL/KsJ-db/db mice with average body weight of 40 ± 5 g were used for the study. Protocol for the study with these animals was approved by the Institutional Animal Ethics Committee (IAEC); Reference no.: IAEC/2015/71 dated 23/07/2015, of the CSIR-Central Drug Research Institute, Lucknow, as per the guidelines of the Committee for the Purpose of Control and Supervision of Experiments on Animals (CPCSEAs), India. The standard animal house conditions with 23 ± 3 °C temperature, 60–70% relative humidity, 300 Lx light at floor level and regular 12 h light/dark cycle, were maintained throughout the experiment. The animals were provided standard pellet diet and drinking water *ad libitum* during the experimental period.

### 4.9. Antihyperglycemic Activity Evaluation in db/db Mice

Mice were held for acclimatization (one week) prior to the experiment, after which animals were randomly divided into two different groups of five animals in each. Group I served as control was treated with vehicle whereas group II was tinosporaside, treated orally once daily with tinosporaside (30 mg/kg) for 15 days. Blood glucose level of each animal was measured daily using a glucometer (Roche, Indianapolis, IN, USA). Oral glucose tolerance test of animals was performed on day 10 and day 15 after an overnight fast. After measuring the baseline glucose level at 0 min, animals were administered with glucose load of 3 g/kg body weight, and glucose levels were measured at 30, 60, 90, and 120 min post-glucose administration [42]. Blood sample was collected from the retroorbital plexus for biochemical analysis. At termination of experiment, animals were sacrificed and samples of skeletal muscle were collected and stored at −80 °C until use.

### 4.10. Biochemical Estimation

Serum samples were prepared by centrifuging blood samples at 3000 rpm for 10 min at 4 °C. Biochemical estimation of triglycerides, cholesterol, and HDL-cholesterol in serum sample was performed using respective biochemical assay kits (Dialab, India) following manufacturer’s instructions. Measurement of serum insulin level was performed using mouse insulin ELISA kit (RayBiotech, Peachtree Corners, GA, USA), as per the manufacturer’s instructions.

### 4.11. Gene expression Analysis

Skeletal muscle samples were homogenized and total RNA was isolated using Trizol reagent. Quality and quantity of isolated RNA was assessed using spectrophotometry and an aliquot of RNA (2 µg) from each sample was reverse transcribed to synthesize cDNA using the High Capacity cDNA Reverse Transcription Kit (Applied Bio systems, Foster, CA, USA), according to the manufacturer’s instructions. Gene expression was analyzed using real-time PCR on PCR Light Cycler 480 System (Roche, Indianapolis, Basel, Switzerland) using a SYBR Premix Ex Taq (ABI-Applied Bio systems, Waltham, MA, USA) according to the supplier’s instructions. Gapdh was used as an internal reference for normalization of relative mRNA expression and fold change was calculated according to (2^−ΔΔCt^) method. Primers sequences used for RT-PCR are provided in Table 1.

### 4.12. Western Blot Analysis

Skeletal muscle samples were lysed in RIPA buffer and centrifuged at 10,000 rpm for 10 min at 4 °C. Supernatant was collected and protein concentration was measured using Lowry assay. For western blotting, equal amount of protein samples was heated in Laemmli sample buffer with 10% β-mercaptoethanol at 75 °C for 10 min and resolved using SDS-PAGE and transferred to PVDF membrane. After blocking, blots were incubated with indicated primary antibodies overnight at 4 °C, followed by washing and incubation with appropriate HRP-conjugated secondary antibodies. Immunoblots were developed using enhanced Chemiluminescence (ECL, Millipore, Burlington, MA, USA) reagent. Densitometric quantification of protein bands was performed using National Institutes of Health (NIH), *Image J* software (version 1.53e, National Institutes of Health, Bethesda, MD, USA), as described previously [43].

### 4.13. Statistical Analysis

All the results are given as mean ± S.E.M. and analyzed using Prism software version 3.0 (Graph Pad software, San Diego, CA, USA). One-way ANOVA was employed for statistical significance, followed by Dunnett’s post hoc test. * *p* < 0.05, ** *p* < 0.01, and *** *p* < 0.001 were used as the significance values.

## 5. Conclusions

The tinosporaside from the *Tinospora cordifolia* augments glucose utilization in skeletal muscle cells following PI3K- and AMPK-dependent signaling mechanisms and exerted antidiabetic effects in db/db mice. Altogether, our findings reveal antidiabetic potential of the tinosporaside, mediated through enhancing the rate of glucose utilization in skeletal muscle via PI3K- and AMPK-dependent signaling mechanisms and can be a potential candidate for the regulation of glucose homeostasis under diabetes.

## Figures and Tables

**Table 1:** Sequence of primers (5′-3′) used for RT-PCR analysis.

Gene	Forward Primer	Reverse Primer
IRS1	AGGAGGAGGGAGGAGAAGG	GAAGAGATCGGGGAAGACG
PI3KCG	CCATGAGGAAACCCAGTGAG	GCGGAGGTTGTCCTCTCTTA
AKT2	GGGCCTGACTCCGAGAAG	CCGCTCCTTATTTATGAACTGG
GLUT4	GACGGACACTCCATCTGTTG	CATAGCTCATGGCTGGAACC
AMPK	CCTTCGGGAAAGTGAAGGT	GAATCTTCTGCCGGTTGAGT
MAPK	TGAAGTTGAACAGGCTCTGG	AATGGCGCTTCAGCAATG
GAPDH	AGCTTGTCATCAACGGGAAG	TTTGATGTTAGTGGGGTCTCG

## Data Availability

Data supporting the reported results will be available with A.K.S. and the corresponding author (R.M.).

## Sample Availability

Sample of the tinosporaside used in the study is available with the R. Maurya (Corresponding author).
